# Global scientific trends on thyroid disease in early 21st century: a bibliometric and visualized analysis

**DOI:** 10.3389/fendo.2023.1306232

**Published:** 2024-01-17

**Authors:** Mingyuan Song, Wei Sun, Qi Liu, Zhongqing Wang, Hao Zhang

**Affiliations:** ^1^ Department of Thyroid Surgery, The First Hospital of China Medical University, Shenyang, China; ^2^ Department of Information Center, The First Hospital of China Medical University, Shenyang, China

**Keywords:** bibliometric, thyroid disease, thyroid cancer, autoimmune thyroiditis, fine needle aspiration, radioactive iodine

## Abstract

**Background:**

Bibliometrics has been used to analyze the literature in the field of thyroid disease studies in the early 21st century, indicating the changes in current international study trends.

**Methods:**

In this study, a bibliometric analysis of data retrieved from the Web of Science (WoS) database was conducted, and the publication trends and thematic evolution in the field of thyroid disease research from January 1, 2000, to November 16, 2022, were analyzed. A total of 69283 articles related to thyroid diseases were evaluated for their characteristics, including annual publication volume, countries, journals, institutions, authors, keywords, and references. VOSviewer was utilized to perform the analysis of co-authorship, co-citation, co-occurrence and descriptive.

**Results:**

The annual publication volume of thyroid disease research literature showed a fluctuating upward trend from 2000 to 2021, exceeding 5,000 articles for the first time in 2021. The United States (16120 counts, 678255 cities) ranks first in terms of publication volume and citation. *Thyroid* (n=3201) and *Journal of Clinical Endocrinology&Metabolism* (n=140399) are the most prolific and cited journals, respectively. The organization with the highest publication volume and citation frequency is Harvard University (1011 counts, 59429 cities), Miyauchi Akira (n=422), Schlumberger, and Martin (n=24839) possess the highest publication volume and citation frequency, respectively. Co-occurrence analysis of 307 keywords with frequencies of more than 20 resulted in 6 clusters (1): Thyroid dysfunction and diseases (2); mechanism of occurrence and development of thyroid cancer (3); autoimmune thyroiditis (4); scope and postoperative management of thyroid surgery (5); fine needle aspiration of thyroid nodules (6); radioactive iodine therapy for thyroid cancer. Active monitoring, thermal ablation, Lenvatinib, and long noncoding RNA refer to the latest keywords. Discussing the six clusters helps scholars to determine the scope and direction of studies.

**Conclusion:**

Over the past two decades, the literature related to thyroid diseases has increased year by year, with closer collaboration between countries, institutions, and authors. In this study, the global trends, research hotspots, emerging subjects, and basic knowledge of literature related to thyroid diseases were respectively elucidated, which will facilitate researchers in this field to seek better development.

## 1 Introduction

Thyroid diseases are most common clinical endocrine system diseases, mainly including hyperthyroidism, hypothyroidism, thyroid nodules and thyroid adenomas, etc. The incidence of thyroid disorders has increased over the past few decades. It’s worth noting that the incidence of thyroid cancer has increased slightly, with PTC growth accounting for the majority of the increase. However, the mortality rate of patients with thyroid cancer remains low, which may be caused by overdiagnosis. Therefore, whether to choose immediate surgery or active surveillance for the treatment of thyroid cancer has become one of the hot topics of discussion. In addition, benign thyroid diseases (e.g., hyperthyroidism and hypothyroidism), are still the most common endocrine diseases worldwide (1, 2). With the deepening of research, research directions and hot spots have also shifted. Up to now, researchers have made significant progress in exploring the mechanisms of thyroid disease, and the diagnosis, treatment, and prevention of thyroid disorders are becoming increasingly personalization. Researches are beneficial for thyroid disease patients to receive more accurate treatment and obtain a better prognosis.

The field of bibliometrics is expanding with the increase in the publication of scientific literature. Bibliometrics is a quantitative analysis of publications. By analyzing the data in the database and using appropriate statistical methods, bibliometrics can be used to evaluate the development state of a certain field over time, which has been an essential component of research quality assessment (3). To date, several bibliometric articles have been published in the direction of thyroid diseases (4, 5), but no comprehensive bibliometric analysis has been performed for all thyroid diseases. This study is the first bibliometric analysis of the whole field of thyroid diseases based on plenty of thyroid disease literature.

By analyzing the number and citation frequency of thyroid disease publications in the early 21st century, this study aims to reveal the publishing trend in research of thyroid disease during this period, identify influential journals, countries, institutions, and authors, and explore the international cooperative network of research, research hotspots, and emerging subjects. Analysis of these data contributes to a vivid exhibitions of shifts in research hotspots and helps researchers focus on areas of interest and significance.

## 2 Methods

This study is based on the Web of Science (WoS) database. As the most commonly used website for obtaining specific literature citations, WoS has historically been the standard for defining citations included in the calculation (6).

**Figure 1:**
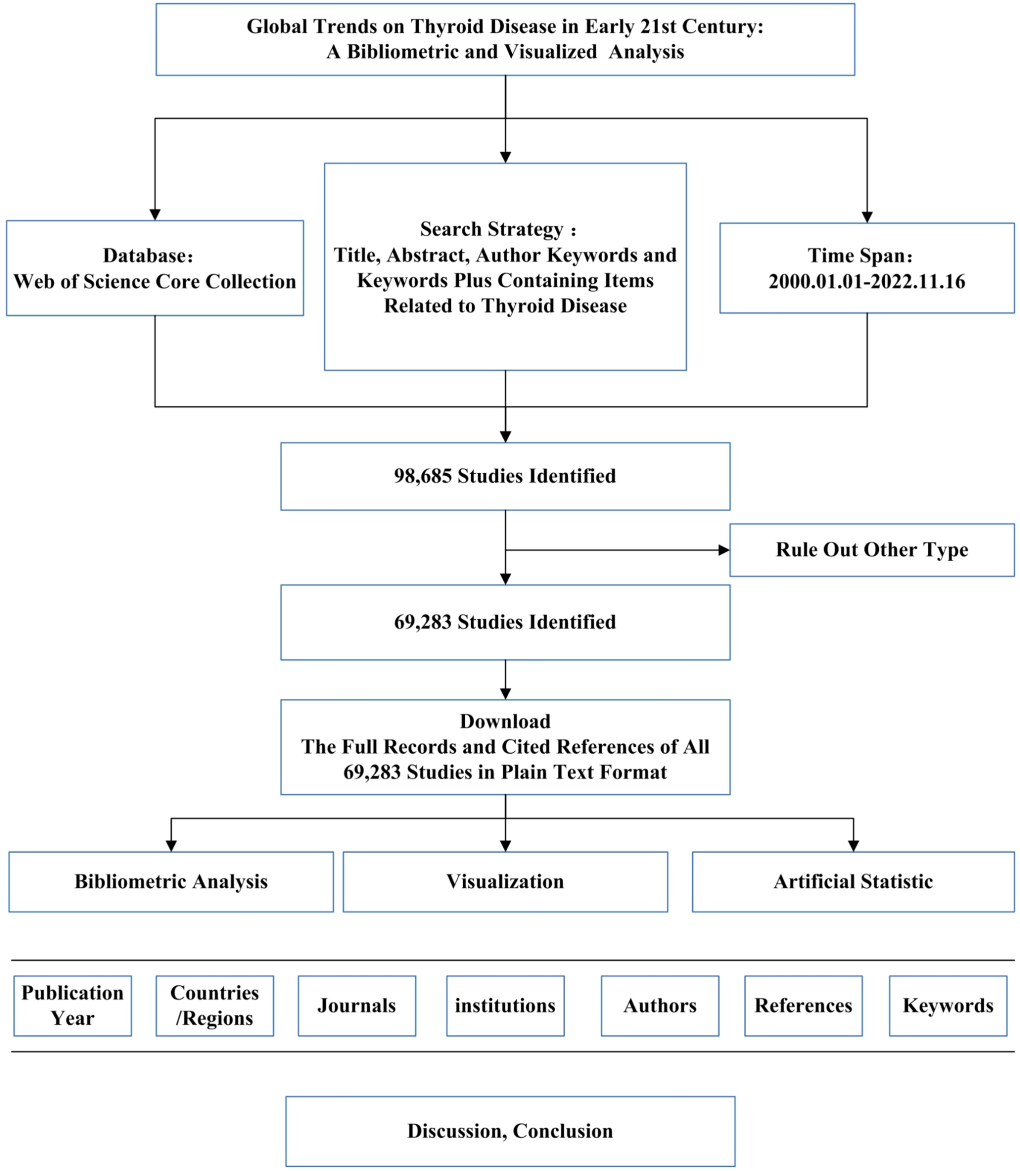
The data collection and retrieval strategy.

As shown in the following figure, data retrieval was conducted on the WoS core collection on November 16, 2022, and the retrieved publications were required to meet the following criteria:

(1)Retrieval strategy: Title, Abstract, Author Keywords, and Keywords Plus Containing Items Related to Thyroid Disease. The retrieval strategy was as follows: TS=(“Thyroid Disease$” or “Euthyroid Sick Syndrome$” or “Sick Euthyroid Syndrome$” or “Non Thyroidal Illness Syndrome$” or “Low T3 Low T4 Syndrome$” or “Low T3 and Low T4 Syndrome$” or “Low T3 High T4 Syndrome$” or “High T4 Syndrome$” or “Low T3 Syndrome$” or “Goiter$” or “Graves$ Disease*” or “Basedow* Disease$” or “Graves Ophthalmopathy” or “Thyroid Eye Disease$” or “Thyroid Associated Ophthalmopath*” or “Dysthyroid Ophthalmopath*” or “Graves Eye Disease$” or “Graves Orbitopathy” or “Myopathic Ophthalmopath*” or “Congestive Ophthalmopath*” or “Edematous Ophthalmopath*” or “Infiltrative Ophthalmopath*” or “Hyperthyroid*” or “Thyrotoxicos*” or “Thyroid Crisis” or “Thyrotoxic Storm” or “Thyrotoxic Crisis” or “Thyroid Storm” or “Hyperthyroxinemia$” or “Thyroid Hormone Resistance” or “Refetoff DeWind DeGroot Syndrome” or “Refetoff Syndrome” or “Generalized Resistance to Thyroid Hormone” or “Hypothyroidism$” or “Thyroid Stimulating Hormone Deficienc*” or “TSH Deficienc*” or “Cretinism” or “Fetal Iodine Deficiency Disorder” or “Myxedema$” or “Lingual Thyroid$” or “Thyroid Dysgenesis” or “Thyroid Hypoplasia” or “Ectopic Thyroid$” or “Thyroid Agenesis” or “Thyroid Nodule$” or “Thyroid Neoplasm$” or “Thyroid Carcinoma$” or “Cancer$ of Thyroid” or “Thyroid Cancer$” or “Cancer$ of the Thyroid” or “Thyroid Adenoma$” or “Thyroiditis” or “Thyroiditides” or “Hashimoto* Disease” or “Hashimoto* Struma” or “Hashimoto* Syndrome$”). Please refer to **Figure 1** and the **Appendix** for details.
(2)Time frame of publication: January 1, 2000, to November 16, 2022.
(3)Only “article” was selected as the article type.

To avoid the impact of database updates, all bibliometric data were downloaded by year on November 16, 2022.

VOSviewer is a classic bibliometric analysis software which is used for building and viewing bibliometric maps. It focuses more on the mapping representation of bibliometric maps and is now widely used for bibliometric analysis and research (7). In this study, Vosviewer version 1.6.18 was used for Bibliometric and Visualized Analysis. Additionally, the citation analysis of journals, the co-authorship analysis of institutions and authors, the co-citation analysis of references, and the co-occurrence analysis of keywords were carried out. A descriptive analysis of publication year, journals, countries, institutions, authors, and references were also performed.

## 3 Results

### 3.1 Annual publication volume

**Figure 2:**
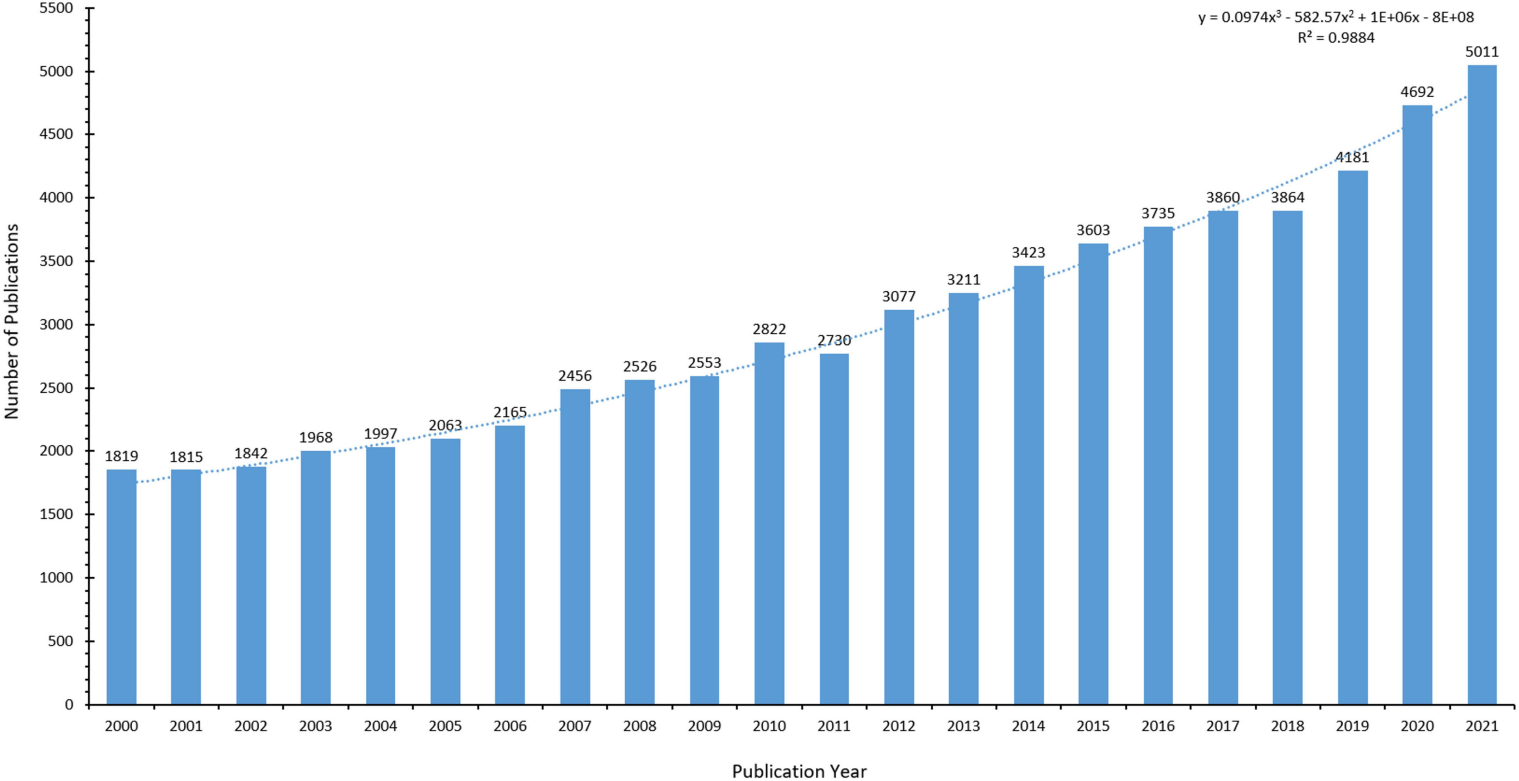
Number of articles on thyroid diseases per year from 2000 to 2021.

A total of 69,283 relevant publications were retrieved, of which 66803 were in English, accounting for 96.42%. The trend figure of annual publication volume from 2000 to 2021 is depicted in **Figure 2** (2022 is not full-year data and not shown in the figure). Linear regression analysis found that the increase in publications per year was statistically significant (P<0.001) (8). The annual publication volume of Thyroid Disease research tends to rise between 2000 and 2021. In particular, there were 1819 publications on thyroid disease research in 2000. In 2021, the number of publications reached the peak of the annual number of publications, which is 2.75 times the number of publications in 2000.

### 3.2 Countries and regions

**Figure 3:**
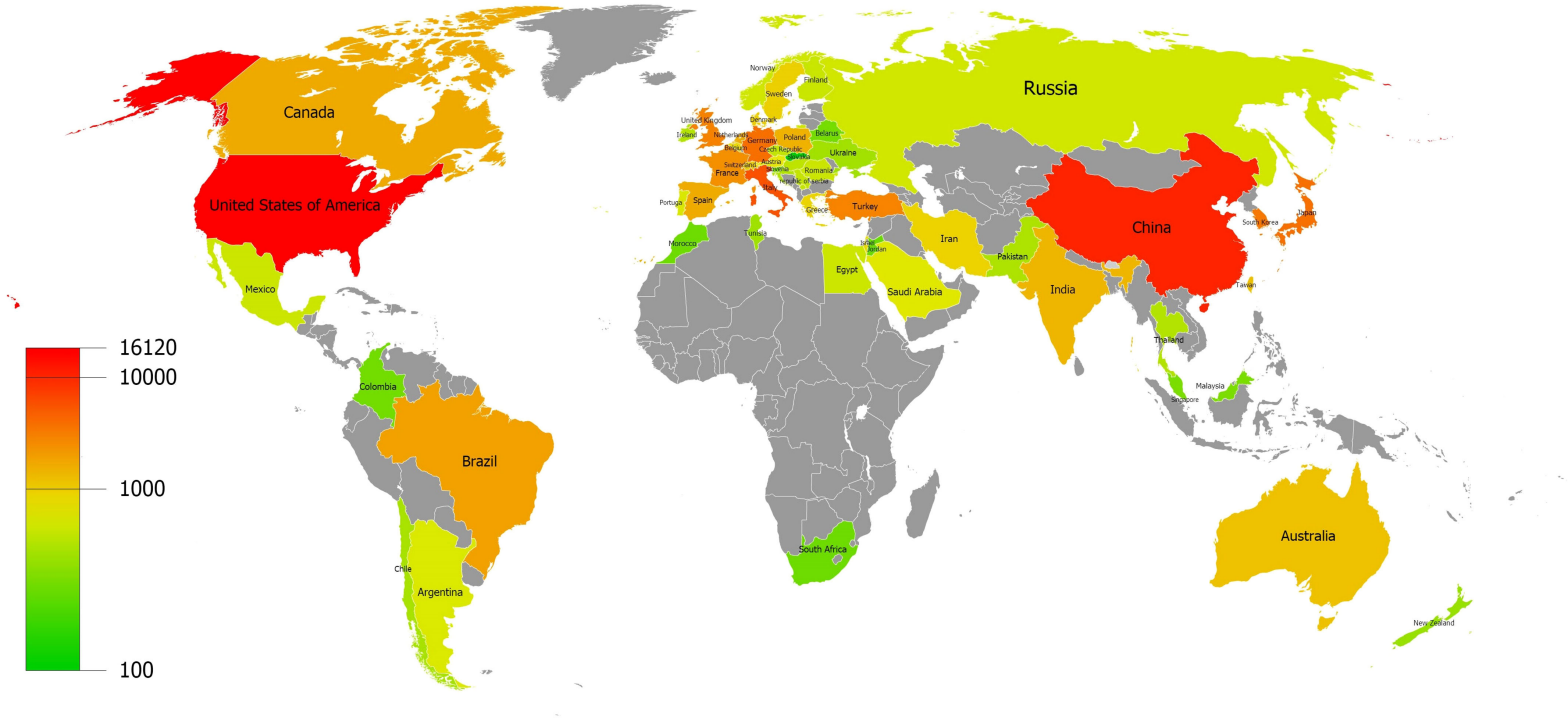
Distribution of countries and regions.

Globally, 175 countries and regions are already involved in thyroid disease research. The threshold for the number of articles issued by countries/regions was set to 100 and 54 high-yield countries/regions in the Thyroid Release research were obtained. The distribution of high-yield countries/regions is described in **Figure 3**. The top ten countries ranked by publication volume are shown in **Table 1**. The United States (16120 counts, 678255 citations) ranks first for both publication volume and citations. This is followed by China (10,565 counts, 141,486 citations) and Italy (6,333 counts, 222,807 citations). Among the top 10 countries, France has the highest average citations (45 avg. Citations). Moreover, China has the highest average publication year, followed by South Korea.

**Table 1:** Top 10 countries in terms of publication volume.

Rank	Country	Counts	Citations	Avg. Citations	Avg. Pub. Year
1	United States of America	16120	678255	42	2012.20
2	China	10565	141486	13	2017.72
3	Italy	6333	222807	35	2012.24
4	Germany	4374	128569	29	2011.45
5	Japan	4317	100976	23	2011.21
6	South Korea	3868	91837	24	2015.19
7	United Kingdom	3341	135132	40	2011.92
8	Turkey	3323	40572	12	2012.91
9	France	2744	124023	45	2011.18
10	Brazil	2131	45430	21	2012.86

### 3.3 Journals

Based on the retrieval results, a total of 4203 journals with publications about thyroid disease research was found. The threshold for journal publication volume was set at 100 articles, and 113 high-yield journals were obtained. These high-yield journals have published 31348 articles, comprising 45.25% of the total publication volume.

**Figure 4:**
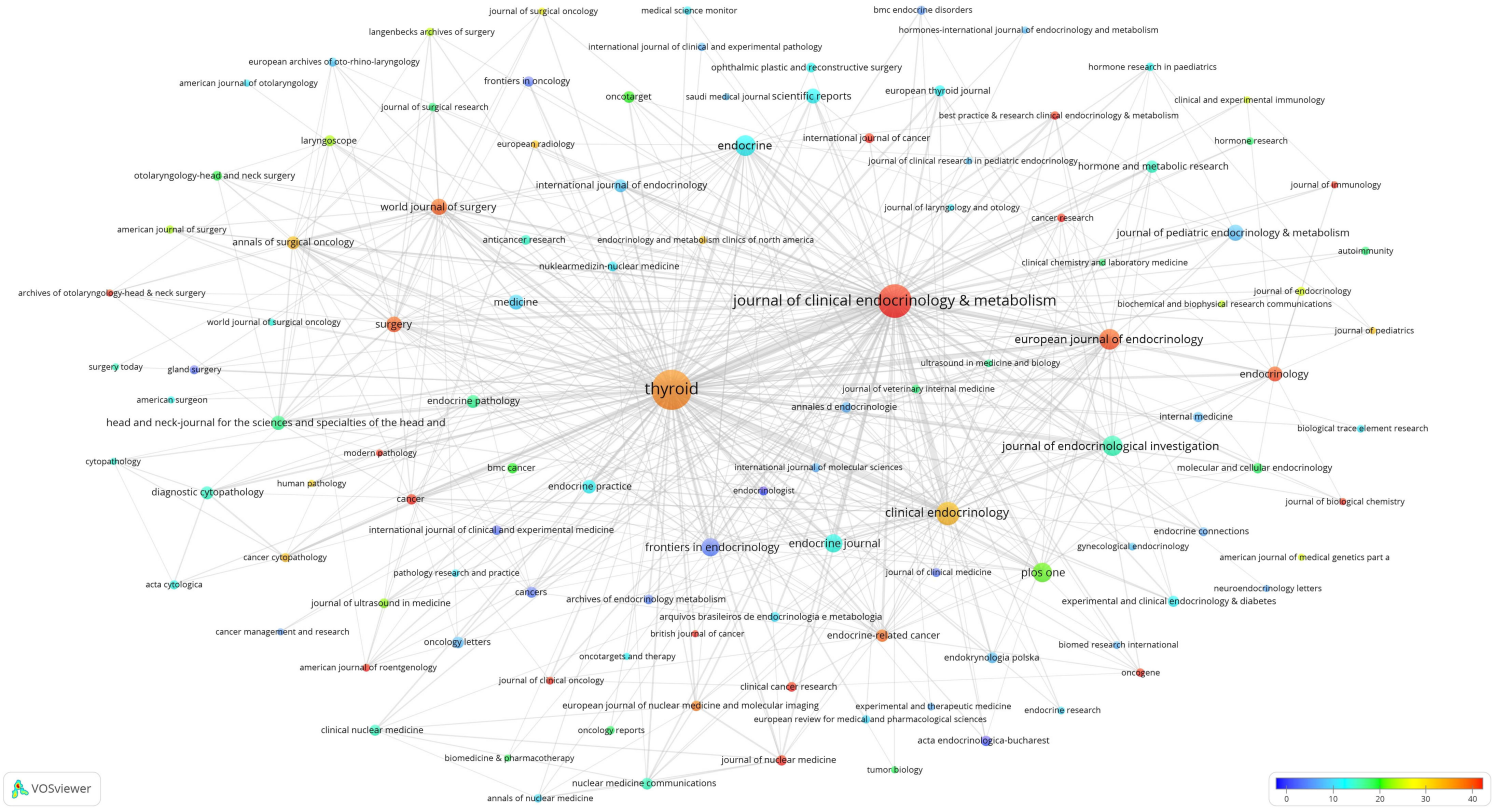
Map of 113 high-yield journals and their citation frequency.

Furthermore, a citation analysis on 113 high-yield journals was conducted and an overlay visualization map was constructed (**Figure 4**). The size of the circle represents the number of publications, and the color range from blue to red denotes the average number of citations from low to high. It can be seen that *Thyroid* (n=3201) is the most prolific journal in this field, followed by the *Journal of Clinical Endocrinology & Metabolism* (n=2285) and *Clinical Endocrinology* (n=1110). The Journal of Clinical *Endocrinology and Metabolism* (n=140399) is the most cited journal overall, followed by *Thyroid* (n=113871) and *Clinical Endocrinology* (n=35716). 14 journals (as shown in red) have an average of more than 40 citations.

### 3.4 Institutions

**Figure 5:**
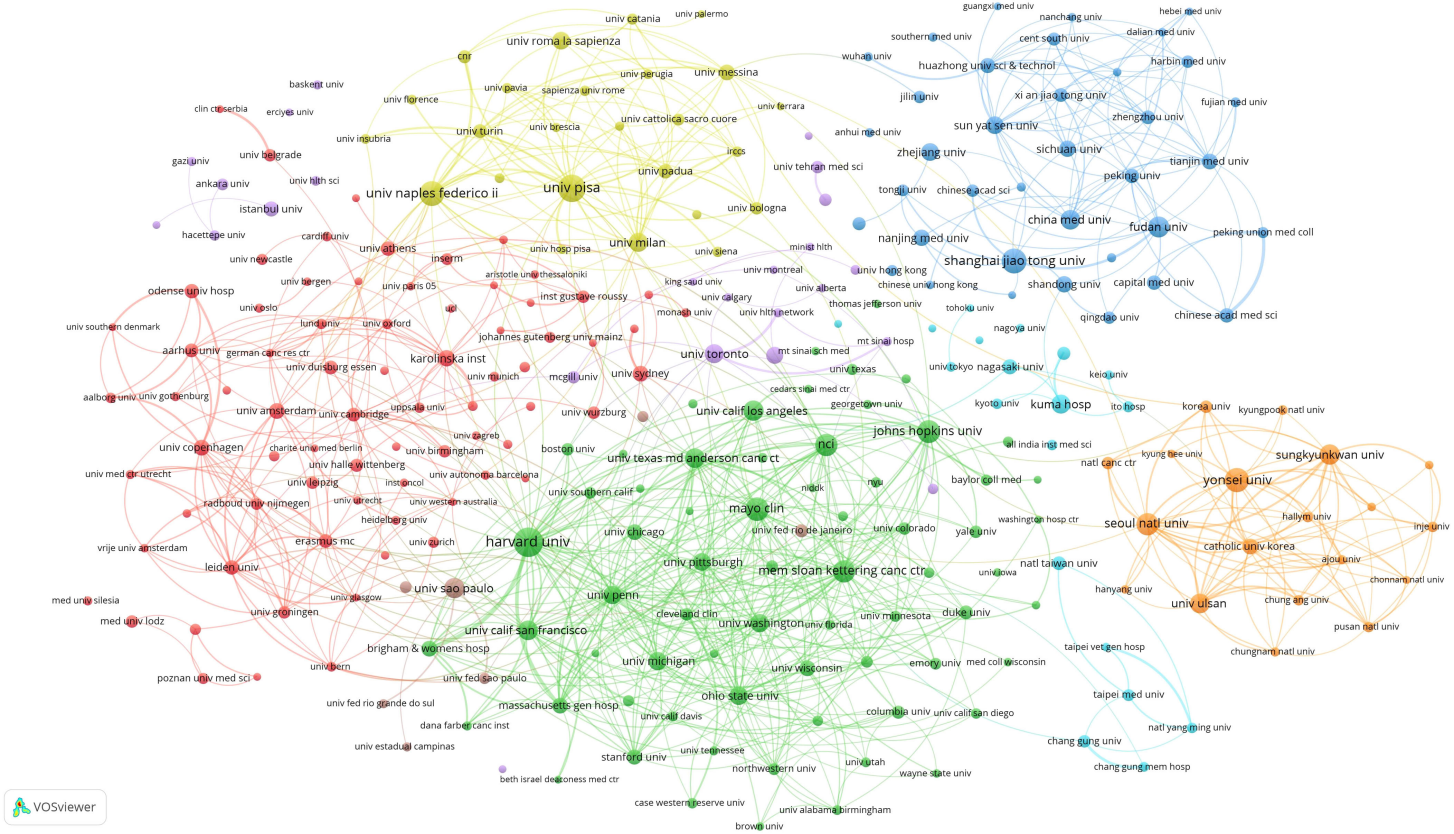
Co-authorship institutional network.

32935 institutions were involved in the publication of thyroid disease papers, and the top 20 institutions by publication volume are shown in **Table 2**. Harvard University (n=1011) is the institution with the highest number of publications, followed by University of Pisa (n=913) and Shanghai Jiao Tong University (n=778). Harvard University(n=59429) is the most frequently cited institution, followed by Memorial Sloan Kettering Cancer Center(n=53014) and University of Pisa(n=44829). Among the top 20 institutions, the Memorial Sloan Kettering Cancer Center and The University of Texas MD Anderson Cancer Center have the highest average citations. Besides, Fudan University has the latest average publication year, followed by Shanghai Jiao Tong University and China Medical University. The threshold for institutional publications was set at 100 and 273 high-yield institutions out of 32,935 were identified. Vosviewer was used to conduct co-authorship analysis of 273 high-yield institutions, all of which were in a co-authorship network, consisting of 8 clusters (**Figure 5**). The red cluster is the largest and consists of 75 institutions. Harvard University has partnerships with 222 high-yield institutions, followed by Johns Hopkins University, with 186 high-yield institutions. Mayo Clinic and The University of Texas MD Anderson Cancer Center both have partnerships with 173 high-yield institutions.

**Table 2:** Top 20 production institutions.

Rank	Institution	Counts	Citations	Avg. Citations	Avg. Pub. Year
1	Harvard University	1011	59429	59	2013.72
2	University of Pisa	913	44829	49	2011.59
3	Shanghai Jiao Tong University	778	10480	13	2017.23
4	University of Naples Federico II	768	30346	40	2010.63
5	Yonsei University	717	18668	26	2014.86
6	Mayo Clinic	680	39744	58	2013.50
7	Johns Hopkins University	645	45392	70	2012.39
8	National Cancer Institute	640	36789	57	2012.59
9	Memorial Sloan Kettering Cancer Center	636	53014	83	2013.66
10	Seoul National University	630	17637	28	2015.48
11	Fudan University	554	7598	14	2017.47
12	Sungkyunkwan University	543	13434	25	2014.92
13	University of California, Los Angeles	529	25033	47	2010.97
14	University Of Sao Paulo	521	15444	30	2012.45
15	University of Ulsan	520	20198	39	2015.34
16	The University of Texas MD Anderson Cancer Center	510	42508	83	2015.45
17	China Medical University	506	9568	19	2016.96
18	University of California, San Francisco	503	27716	55	2011.89
19	University of Milan	486	20422	42	2013.03
20	Kuma Hospital	482	13664	28	2011.19

### 3.5 Authors

A total of 248011 authors participated in the publication of *Thyroid Disease*, and the top 20 authors ranked by publication volume are in **Table 3**. Miyauchi, Akira (n=422) is the author with the highest number of publications, followed by Tuttle, R. Michael (n=236) and Ito, Yasuhiro (n=234). Schlumberger, Martin (n=24839) is the most frequently cited author, followed by Tuttle, R. Michael (n=22632) and Elisei, Rossella (n=15474). On average, Schlumberger, Martin (n=117) is the most frequently cited, followed by Tuttle, R. Michael (n=96) and Elisei, Rossella(n=74). Among the top 20 authors, Baek, Jung Hwan has the latest average publication year, followed by Shan, Zhongyan, and Teng, Weiping.

**Table 3:** Top 20 authors in terms of publication volume.

Rank	Author	Counts	Citations	Avg. Citations	Avg. Pub. Year
1	Miyauchi, Akira	422	11677	28	2010.97
2	Tuttle, R. Michael	236	22632	96	2012.58
3	Ito, Yasuhiro	234	8242	35	2010.60
4	Dralle, Henning	233	11436	49	2009.48
5	Schlumberger, Martin	213	24839	117	2011.16
6	Elisei, Rossella	209	15474	74	2013.62
7	Shong, Young Kee	199	7475	38	2015.11
8	Hegedus, Laszlo	198	8573	43	2012.12
9	Pinchera, A	195	12508	64	2005.84
10	Vitti, Paolo	191	7847	41	2012.37
11	Kim, Tae Yong	190	5865	31	2015.56
12	Teng, Weiping	188	4774	25	2015.94
13	Kim, Eun-Kyung	182	6636	36	2013.39
14	Miya, Akihiro	178	6111	34	2011.08
15	Kim, Won Bae	177	5619	32	2015.10
16	Basolo, Fulvio	176	10197	58	2012.63
17	Shan, Zhongyan	176	4568	26	2016.03
18	Kwak, Jin Young	175	5499	31	2014.18
19	Baek, Jung Hwan	175	6369	36	2016.32
20	Chen, Herbert	173	7220	42	2012.53

**Figure 6:**
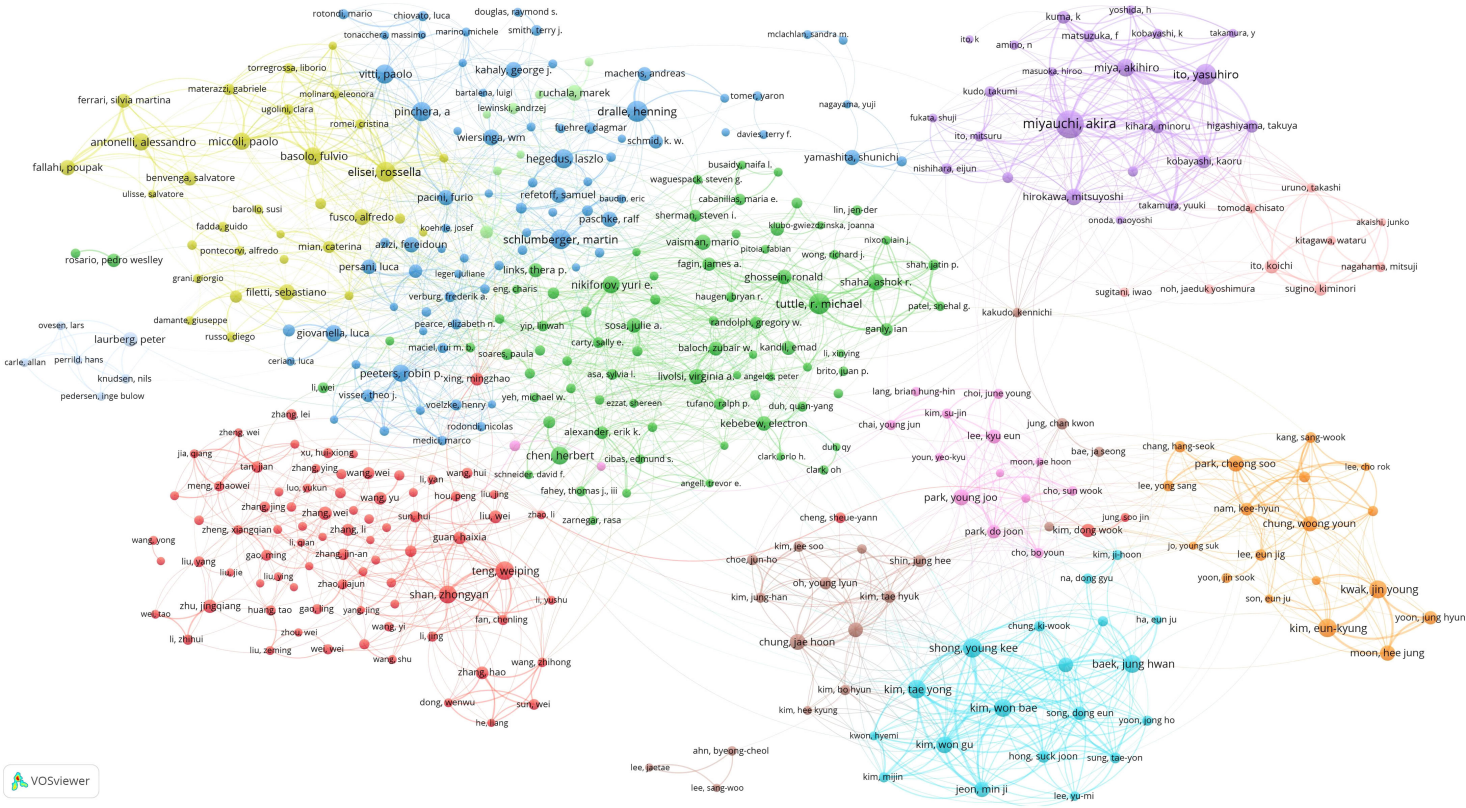
Co-authorship network.

The author publication threshold was set at 40 articles and 397 highly productive authors from 248011 authors were identified. As demonstrated in **Figure 6**, a co-authorship analysis on 397 high-yield authors was carried out using Vosviewer. 388 of the 397 high-producing authors formed the largest network of co-authors, consisting of 12 clusters. Tuttle, R. Michael has partnerships with 89 high-yield authors. Secondly, Elisei, Rossella has partnerships with 77 high-yield authors, while Fugazzola, Laura has partnerships with 71 high-yield authors.

### 3.6 Reference

**Figure 7:**
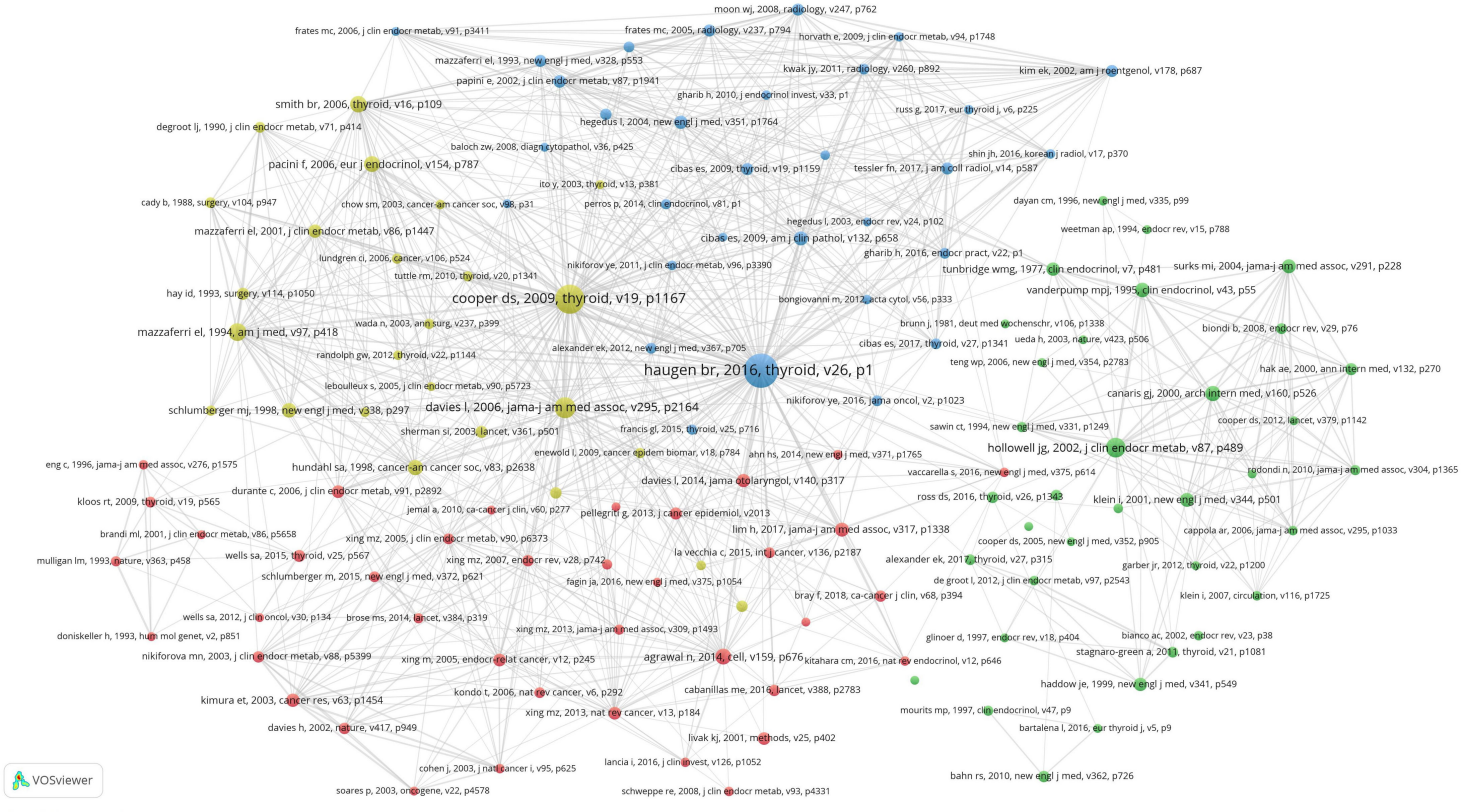
Citation analysis of references.

A total of 852113 references were cited in 69283 publications, of which 852113 references appeared a total of 2282274 times, with an average of 33 references per publication. The threshold of reference citations was set to 300 times and 130 highly cited references from 852113 reference articles were identified. As shown in **Figure 7**, a co-citation analysis on 130 highly cited references was conducted using Vosviewe.

The top five articles cited are as follows: Haugen br, 2016, thyroid, v26(citation frequency:4549), Ooper ds, 2009, thyroid, v19(citation frequency:3306), Davies l, 2006, jama-j am med assoc, v295(citation frequency:1700), Hollowell jg, 2002, j clin endocr metab, v87(citation frequency:1454),Mazzaferri el, 1994, am j med, v97(citation frequency:1253).

### 3.7 Keywords analysis

**Figure 8:**
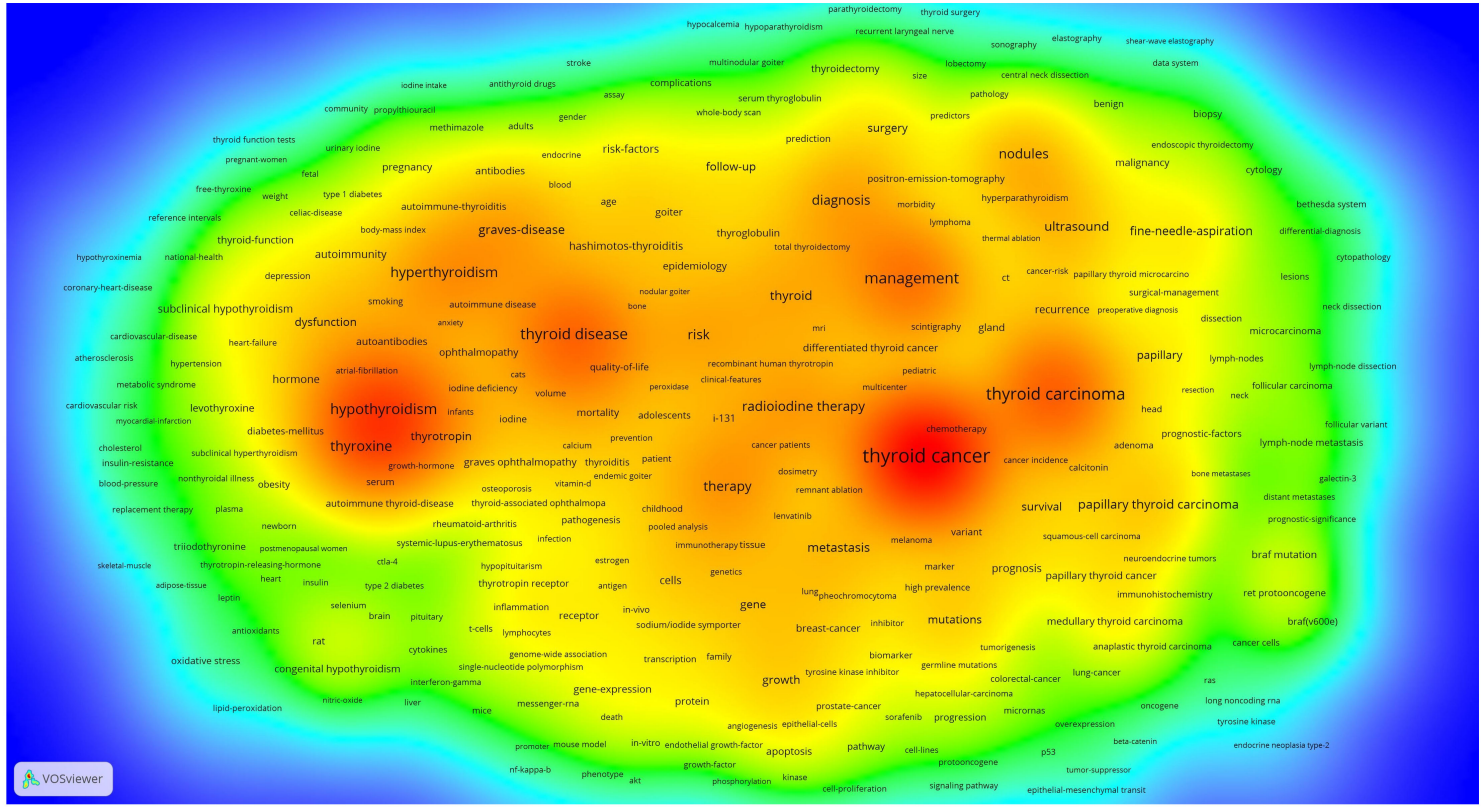
Co-occurrence heat map.

**Figure 9:**
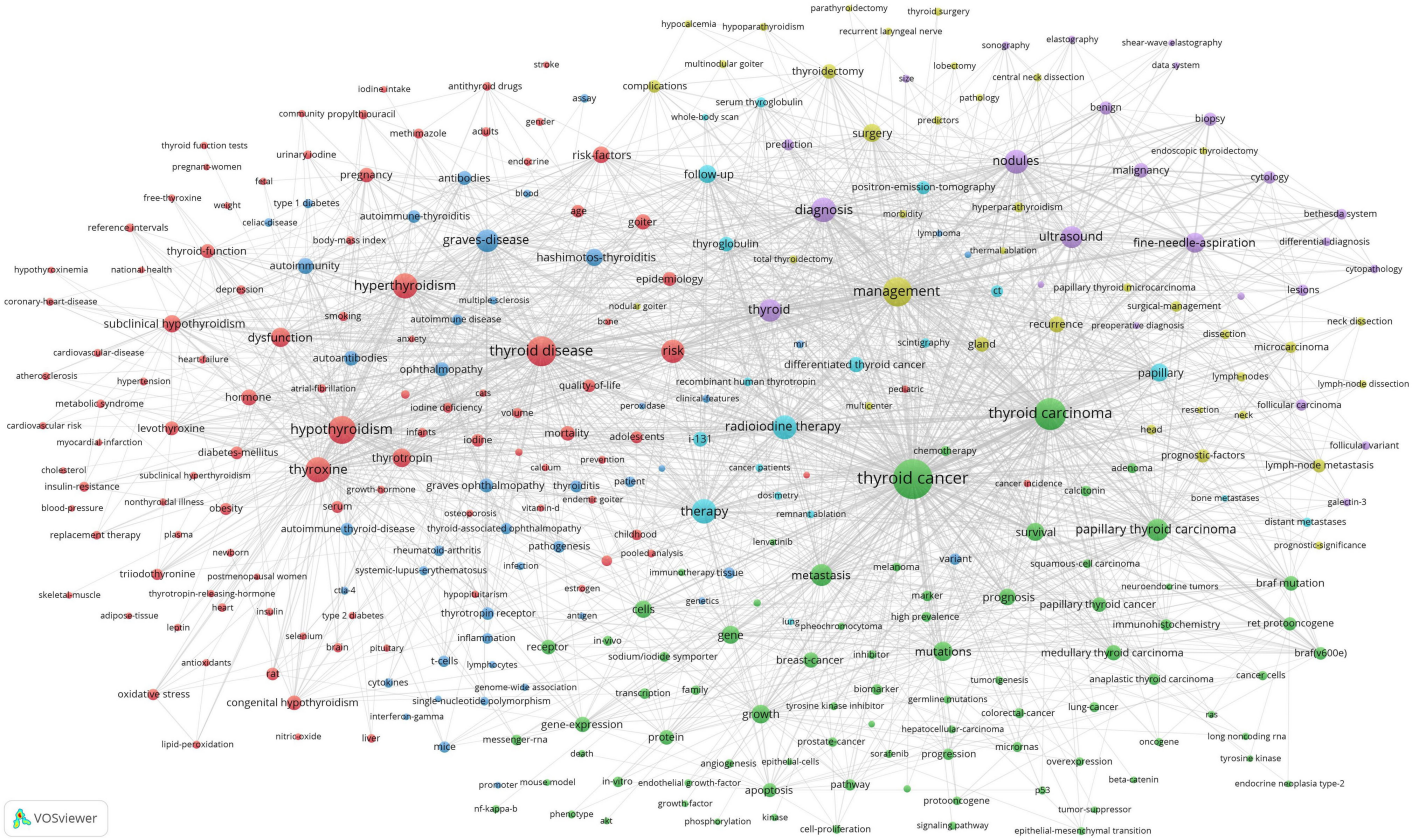
Co-occurrence network map.

There are 92426 keywords in 69283 articles. The threshold of high-frequency keywords was set to 20, of which 307 keywords were selected. A Co-occurrence analysis was conducted on 307 high-frequency keywords and a co-occurrence network map was constructed. At the same time, we also built heat maps for a clearer and more intuitive presentation of the results (**Figure 8**). The co-occurrence analysis of 307 high-frequency keywords formed 6 clusters, represented by different colors (**Figure 9**). The red cluster consists of 102 keywords and is the largest cluster. Cluster1(red), Cluster2(green), Cluster3(blue), Cluster4(yellow), Cluster5(purple), and Cluster6(light blue)focus on the subject respectively: 1) Thyroid dysfunction and diseases; 2) mechanism of occurrence and development of thyroid cancer; 3) autoimmune thyroiditis; 4) scope and postoperative management of thyroid surgery; 5) fine needle aspiration of thyroid nodules; and 6) radioactive iodine therapy for thyroid cancer.

**Figure 10:**
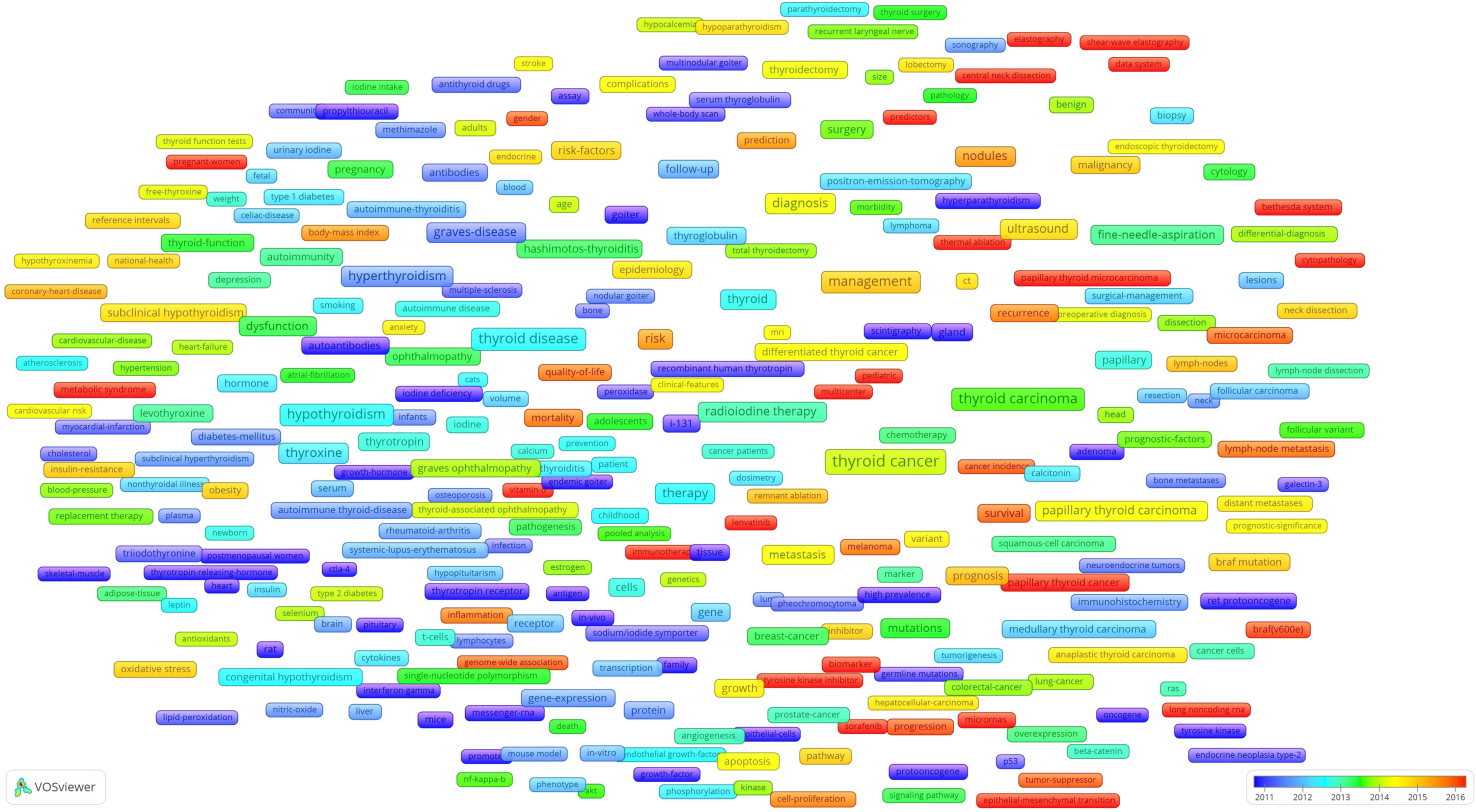
Co-occurrence overlay map.

An Overlay map of 307 high-frequency keywords was constructed (**Figure 10**). The color of each node in the graph indicates the average year of publication for that node and the year of publication changes from blue to red.

## 4 Discussions

### 4.1 Overall trend

The data shows that from 2000 to 2021, the overall annual number of publications in thyroid disease research has increased each year for the past 21 years, although the number of publications fluctuated slightly in some years. The United States ranks first in both publication volume and citation frequency, and is the leader in this field. Interestingly, in recent years, there has been a rapid increase in research on thyroid disorders in China and Korea, which may be related to the increased incidence and increased interest of researchers (9, 10). In the top 20 institutions concerning publication volume, Harvard University is the most published and most cited institution, collaborating with 222 high-yield institutions and serving as a global hub for research institutional collaboration. Of the 397 high-yield authors, 97% appeared in the collaborative network, suggesting a high level of collaboration among high-yield authors. Among them, Tuttle, R. Michael, Elisei, Rossella, and Fugazzola, Laura, et al. have particularly close collaborations, forming a close cooperative relationship.

### 4.2 Research hotspots

#### 4.2.1 Cluster1(red): thyroid dysfunction and diseases

Hyperthyroidism and hypothyroidism are the most common disorders of thyroid dysfunction. Several factors have been found to contribute to thyroid dysfunction, including the environment, genetic susceptibility, micronutrients (iodine, selenium), infections, and immune system defects (11, 12). In addition, a combination of TSHR and TSHR autoantibodies, as well as amiodarone, a drug structurally similar to thyroid hormones for the treatment of arrhythmia, may also affect thyroid function (13, 14). In the past, thyroid dysfunction was thought to increase the risk of certain diseases, such as cardiovascular disease (15), Psychiatric Disorders (16), renal dysfunction (17), and changes in bone mineral density (18). However, new studies reveal that some of these diseases may not be associated with thyroid dysfunction (19, 20). Interestingly, researchers have found that early thyroid dysfunction was closely related to adrenal gene expression and hormone secretion (21). As for treatment, the cancer risk after radioactive iodine treatment for hyperthyroidism is low (22). Total thyroidectomy is a reliable method for the treatment of hyperthyroidism, without significant differences in the incidence of postoperative complications compared to patients with normal thyroid function (23). Nevertheless, children with hyperthyroidism are more likely to suffer from hoarseness and difficulty concentrating after surgery than adults (24). Using levothyroxine in the treatment of subclinical hypothyroidism remains controversial (25). The risks brought by long-term treatment cannot be ignored (26), and it is necessary to prevent excessive treatment from causing harm to patients as much as possible (27). Therefore, careful implementation of individualized treatment is the most beneficial option for patients, and specific treatment protocols still need to be validated by researchers through large-scale randomized trials.

#### 4.2.2 Cluster2(green): mechanism of occurrence and development of thyroid cancer

While the mortality rate has remained relatively stable, the incidence rate of thyroid cancer has increased in recent years, which is probably due to the increase in examinations and the emergence of new technologies (28, 29). The mechanism of occurrence and development of thyroid cancer has been a focus of researches. Multiple molecular biomarkers exist in TC, among which RAF-MEK-ERK signaling levels in the MAPK pathway are involved in cancer cell proliferation, differentiation, and development. Among the three RAF subtypes, BRAF is the most common mutation type (30). When the MAPK pathway is activated in TC, cancer cells exhibit stronger invasiveness (31). As one of the most common activation pathways, the PI3K/AKT pathway, including various signaling complex proteins (32), plays an important role in the diagnosis, prognosis, and treatment of TC (33). Targeting PI3K/AKT pathway has become one of the most effective cancer treatments (34). PD-1 is a transmembrane protein expressed in immune cells (35), and its expression in tumors can lead to tumor recurrence and metastasis (36, 37). PD-L1 expression in TC is associated with lower survival rates (38), and immunotherapy targeted PD-1/PD-L1 is increasingly being elucidated (39). Epithelial-mesenchymal transition (EMT) is an important link in cancer progression, which can lead to the invasion and migration of cancer cells (40). In thyroid cancer, genetic changes in EMT-related genes and transcription factors can lead to TC proliferation, migration, and invasion (41). Non-coding RNA (ncRNA) also has an impact on the development of TC. Multiple Long non-coding RNA (lncRNA)s, miRNAs, and circRNAs have been found to play key roles in the occurrence, development, and differentiation of TC (42). The studies on these mechanisms assist in understanding the conditions of drug resistance and discovering new strategies for the treatment of TC.

#### 4.2.3 Cluster3(blue):autoimmune thyroiditis

Autoimmune thyroid disease (AITD) mainly includes Hashimoto’s thyroiditis and Graves’ disease, both of which are caused by lymphocyte infiltration and autoantibody production, leading to immune system dysfunction and then attacks the thyroid gland (43). It is well known that T lymphocytes are the main infiltrating cells in AITD, causing autoimmunity through the production of inflammatory factors. Regulatory B cells have also been found to regulate AITD through inflammatory factors (44). The role of cytokines and chemokines in AITD is gradually being elucidated. Cytokines released from lymphocytes induce the activity of immune cells and contribute to the regulation of the immune response by the body. Interestingly, chemokines in AITD (e.g., CXCL10), may be involved in the occurrence of tumor-related inflammation (45). TgAb and TPOAb are sensitive biomarkers of AITD, and their levels are influenced by various factors and reflect thyroid function (46). Recently, the role of microbiota in AITD has aroused researchers’ interest. It was found that changes in microbiota and diversity of composition are widespread in AITD and may lead to changes in TPOAb levels (47, 48). In addition, the body also affects the development of AITD by releasing exosomes. AITD patients overexpress certain inflammatory factors through exosomes, leading to an increase in serum TPOAb and TgAb levels, thereby affecting the occurrence and development of AITD (49, 50). Myoinositol (Myo) has also been found to have beneficial effects on AITD (51). The emergence of AITD is accompanied by changes in various pathogenic factors. The mechanisms of immune system disorders leading to thyroid immune attack are still under investigation, and researchers need to carefully test more new therapeutic targets.

#### 4.2.4 Cluster4(yellow): scope and postoperative management of thyroid surgery

The scope of thyroid surgery needs to be considered by several factors, including but not limited to tumor invasiveness, risk of recurrence, and changes in quality of life. Despite still being controversial, prophylactic central lymph node dissection is still popular among young, low-risk patients (52). The most important complications related to thyroid surgery are recurrent laryngeal nerve injury and postoperative hypoparathyroidism, both of which can affect a patient’s life quality. These complications cause symptoms that may be temporary or permanent. Hence, doctors should carefully assess the potential risks before surgery. The most common modality of laryngeal return nerve injury is traction (53). Using intraoperative nerve monitoring can reduce recurrent laryngeal nerve injury (54), and continuous intraoperative nerve monitoring is most effective in preventing vocal cord paralysis (55). Permanent hypoparathyroidism is rare, with magnesium deficiency (56), gene mutation (57), and dyslipidemia (58) as potential contributing factors. The most common cause in clinical practice is surgery, and the probability of postoperative occurrence is related to the scope of surgery and professional knowledge (59, 60). Although there are different surgical options for thyroid surgery (61), an increasing number of patients express dissatisfaction with the outcome (62). Therefore, more factors should be taken into consideration when considering treatment options for patients, and new treatment methods should be actively developed.

#### 4.2.5 Cluster5(purple): fine needle aspiration of thyroid nodules

Thyroid nodules have a high incidence in the population, and even with a slight increase in the incidence of thyroid cancer, the likelihood of being a malignant nodule remains low (63). At the same time, nodules have been found to have some characteristics of malignant tendency. For example, the risk of malignancy is higher in middle-lobe-thyroid nodules than in other sites (64). Besides, the malignant risk of spherical nodules is also relatively high (65), so the management and treatment of this kind of patients should not be underestimated. Fine needle aspiration biopsy is the most reliable way to judge the type of thyroid nodules. This method is simple and reliable, which can be employed to improve the diagnostic accuracy of uncertain nodules through ultrasound guidance (66). Although there may be few complications, it is generally safe and effective (67). However, there is still uncertainty regarding the results of fine needle aspiration in thyroid follicular cell carcinoma, follicular thyroid tumor, and follicular variation in papillary thyroid cancer (68). Fortunately, Raman Spectroscopy and molecular diagnosis can be adopted to improve the diagnostic probability of these nodules and avoid unnecessary surgery (66, 69). In addition, the results of continuous ultrasound monitoring can also be used as a criterion to determine the timing of surgery (70). Radiofrequency ablation can be used as a means to treat uncertain thyroid nodules (71). In short, most thyroid nodules are benign, and fine needle aspiration, as the most commonly used diagnostic tool, provides the most definitive diagnostic information.

#### 4.2.6 Cluster6(light blue): radioactive iodine therapy for thyroid cancer

DTC is the most common endocrine tumor and its incidence is increasing year by year, but the mortality is expected to remain unchanged (28). The prognosis of DTC is excellent, depending on the revision of the diagnosis and treatment strategy (72). Radioactive iodine-131 therapy is a common adjuvant treatment for patients with DTC after total thyroidectomy, mainly for the ablation of residual thyroid tissue to reduce disease recurrence and leading to specific mortality (73). Nevertheless, there are also some controversies on RAI, such as the selection of RAI dosage for low-risk thyroid cancer patients (74). It was found that the risk of developing a second primary malignancy increases when the cumulative RAI dose is greater than 150 mCi (75). For patients with DTC with tumor diameters greater than 1 cm, postoperative iodine-131 therapy remains a reasonable option (76). Furthermore, delayed treatment in patients with low-risk and lower-Intermediate risk DTC does not seem to change the final outcome (77). Therefore, additional clinical factors should be considered when using RAI in low-risk and lower-Intermediate risk patients. Unfortunately, RAI is ineffective in the treatment of some radioactive iodine-refractory DTC, such as recurrent or metastatic TC (78). Recent studies have verified that Cabozantinib and a novel molecular inhibitor [177Lu]Lu-DOTAGA.(SA.FAPi)2 may produce a safe and effective treatment for RR-DTC (79, 80). In brief, RAI therapy remains an old but mainstream approach for the treatment of patients with DTC., and higher quality studies are needed to clarify the unsolved problems.

### 4.3 Keywords with relatively new average publication years and emerging subjects

In recent years, there has been an increasing interest in Active Surveillance (AS), mainly due to the excellent postoperative performance of patients with papillary thyroid microcarcinoma (PTMC). AS was initially a prospective study that first suggested that patients with PTMC had a relatively low probability of clinical progression. For patients without high-risk features, regular observation is an option, and it is not too late to undergo surgery when the tumor evolve (81). Subsequently, researchers from multiple developed countries conducted experiments, indicating that this method was only effective for low-risk PTMC and that surgery remains the preferred treatment option for high-risk patients (82–84). In addition, recent studies have demonstrated that AS has a lower incidence of adverse reactions and recurrence rate compared to conversion and immediate surgery, and AS can be the first-line treatment of choice for PTMC (85). As a management strategy, it has been endorsed by several organizations, including the *Japan Association of Endocrine Surgery and the American Thyroid Association* (86–89). Nevertheless, there are also many problems in the management of AS, such as long follow-up times, the limited number of surgeries, and difficulties in specimen collection. Follow-up strategies and timing of interventions for AS require more evidence to confirm its long-term safety. In general, AS is an effective alternative strategy to surgery for low-risk PTC. Currently, concerns about the effectiveness and safety of AS are diminishing, and various versions of the guidelines recommend the implementation of AS.

Thermal Ablation (TA) is a treatment option for PTMC patients in addition to AS and immediate surgery and can significantly reduce the nodule volume, but takes a long time to achieve nodule volume reduction (90, 91). Surgery, as the standard treatment for PTC patients, is the only method to remove the primary lesion and obtain accurate staging and risk stratification. Several studies comparing the effectiveness and safety of TA and immediate surgery have shown no tumor recurrence or distant metastases with both treatments. However, the probability of complications in TA is significantly lower than that in the surgical group, and TA can reduce the probability of hypothyroidism in patients. Besides, patients will have significantly reduced surgical time, recovery time, and hospitalization time (92). The direct comparison between TA and AS currently lacks feasibility. It has been established that TA can address patient anxiety during follow-up and thus avoid surgical delays. Moreover, after five years of follow-up, patients did not develop tumor recurrence, lymph node metastasis, or distant metastases (93, 94). However, TA is not indicated for all types of thyroid nodules, and occasionally incomplete nodal response and local regeneration may occur. At this point, repeat ablation or surgical treatment may be required, which may make the procedure more difficult (95, 96). Hence, prospective studies with larger samples and longer follow-up times are needed to demonstrate its efficacy. Nevertheless, TA can serve as an alternative for low-risk PTMC patients (97).

Anaplastic thyroid carcinoma and metastatic poorly differentiated thyroid carcinoma are highly invasive malignant tumors. Furthermore, despite the availability of several treatments for iodine-refractory thyroid cancers, the overall survival rate remains unsatisfactory, especially in patients with ATC, with a median survival of only 3-5 months (98). The treatment of such tumors with poor prognosis has always been a focus of researchers’ attention. Lenvatinib is an oral, multitargeted tyrosine kinase inhibitor of VEGFR1, VEGFR2, and VEGFR3; FGFR1, FGFR2, FGFR3, and FGFR4 (99), and its role in RR-DTC has been proved in many countries (100). However, the therapeutic efficacy of ATC remains controversial (101, 102), and it leads to a decrease in quality of life (103). Recently, the combination of lenvatinib and pembrolizumab has achieved encouraging results in the efficacy of ATC/PDTC patients (104), and a larger sample is still needed to monitor the therapeutic effect of the combination of both.

Investigating the mechanisms of thyroid cancer occurrence and development may facilitate new diagnostic and therapeutic biomarkers. LncRNA has been identified as a biomarker for several cancers (105). It is highly expressed in PTC and promotes the growth and metastasis of tumor cells (106). Several lncRNAs, such as MALAT1, BANCR, and PTCSC3, have been identified to promote the proliferation and metastasis of thyroid cancers (107–109). In addition, lncRNA has also been associated with lymph node metastasis (110), and further studies are needed to elucidate the remaining unknown functions of lncRNA. This biomarker provides new evidence for individualized therapy and molecular diagnosis.

## 5 Conclusions

The field of thyroid diseases developed rapidly in the early 21st century. Over the past 20 years, the number of publications has been increasing year by year. The United States is the leader in this field, and China and South Korea are developing rapidly. In addition, Harvard University is the center of global research institutions, and there is close cooperation between institutions and authors around the world, especially among high-yield authors. More importantly, six major research themes were discovered by analyzing keywords. As well as active surveillance, thermal ablation and the development mechanism and treatment of thyroid cancer are current research hotspots.

## 6 Strengths

To our knowledge, this study is the first bibliometric analysis of the entire field of thyroid disorders based on a large body of literature on thyroid disorders. The analysis of early 21st-century publications enabled the visualization of thyroid disorders, including publication trends, global collaborations, and research hotspots. These bibliometrics can identify new subjects and frontiers for future research in the field of thyroid diseases.

## 7 Limitations

As with other bibliometric studies, there are certain limitations in this study.

Although there is a lot of literature available in the Web of Science database, there is still some literature that is not included in the Web of Science database, which may lead to biased results.

The statistical results may be biased due to irregularities in the writing of author and institution names.

## Data availability statement

The original contributions presented in the study are included in the article/**Supplementary Material**. Further inquiries can be directed to the corresponding authors.

## Author contributions

MS: Conceptualization, Data curation, Investigation, Writing – original draft. WS: Data curation, Writing – original draft. QL: Data curation, Writing – original draft. ZW: Conceptualization, Investigation, Supervision, Writing – review & editing. HZ: Conceptualization, Investigation, Supervision, Writing – review & editing.
